# Improved image quality in CT pulmonary angiography using deep learning-based image reconstruction

**DOI:** 10.1038/s41598-024-52517-2

**Published:** 2024-01-30

**Authors:** Ann-Christin Klemenz, Lasse Albrecht, Mathias Manzke, Antonia Dalmer, Benjamin Böttcher, Alexey Surov, Marc-André Weber, Felix G. Meinel

**Affiliations:** 1 Institute of Diagnostic and Interventional Radiology, Pediatric Radiology and Neuroradiology, University Medical Centre Rostock, Schillingallee 36, 18057 Rostock, Germany; 2 Department of Radiology, Mühlenkreiskliniken Minden, Ruhr-University Bochum, Bochum, Germany

**Keywords:** Medical research, Respiratory signs and symptoms, Cardiovascular diseases, Respiratory tract diseases

## Abstract

We investigated the effect of deep learning-based image reconstruction (DLIR) compared to iterative reconstruction on image quality in CT pulmonary angiography (CTPA) for suspected pulmonary embolism (PE). For 220 patients with suspected PE, CTPA studies were reconstructed using filtered back projection (FBP), adaptive statistical iterative reconstruction (ASiR-V 30%, 60% and 90%) and DLIR (low, medium and high strength). Contrast-to-noise ratio (CNR) served as the primary parameter of objective image quality. Subgroup analyses were performed for normal weight, overweight and obese individuals. For patients with confirmed PE (n = 40), we further measured PE-specific CNR. Subjective image quality was assessed independently by two experienced radiologists. CNR was lowest for FBP and enhanced with increasing levels of ASiR-V and, even more with increasing strength of DLIR. High strength DLIR resulted in an additional improvement in CNR by 29–67% compared to ASiR-V 90% (p < 0.05). PE-specific CNR increased by 75% compared to ASiR-V 90% (p < 0.05). Subjective image quality was significantly higher for medium and high strength DLIR compared to all other image reconstructions (p < 0.05). In CT pulmonary angiography, DLIR significantly outperforms iterative reconstruction for increasing objective and subjective image quality. This may allow for further reductions in radiation exposure in suspected PE.

## Introduction

Pulmonary embolism (PE) is the third common cardiovascular disease in Europe and North America^1,2^ and CT pulmonary angiography (CTPA) is the most commonly used imaging modality for diagnosis of PE^3,4^. The increasing use of CT scans has raised concerns regarding the long-term risks of radiation exposure^5–7^. Advances in CT acquisition technique including tube current modulation and patient-specific tube voltage selection have already significantly reduced the radiation exposure from CT pulmonary angiography^8^ but are limited by decreasing image quality and increasing image noise at lower dose.

Compared to filtered back projection (FBP) as the historical standard, iterative reconstruction techniques have shown great potential to decrease radiation dose requirements without impairing the image quality as they can substantially decrease image noise in CT pulmonary angiography^9–14^. Iterative reconstruction is a collective term for various algorithms that reduce noise and improve image quality through cyclic image processing in the raw data and/or image data domain^15^. In contrast, the more recent technique of deep learning-based image reconstruction (DLIR) is based on convolutional neural networks trained on a large amount of corresponding high- and low-dose data^16,17^.

Previously, DLIR has been shown to improve image denoising and increase image quality beyond the levels of achieved with iterative reconstruction techniques in various cardiovascular CT applications including coronary CTA^18–23^, CT for planning of transcatheter aortic valve repair^24,25^, CT of the aorta^26^, head and neck CT angiography^27^ and triple-rule-out CT^28^. There are very limited data about DLIR in CTPA despite this being one of the most commonly performed cardiovascular CT examinations. So far only one relative small study previously investigated DLIR in CTPA and reported a significant potential for image quality improvement and noise reduction^29^.

Therefore, the purpose of this study was to investigate the effect of various levels of DLIR reconstructions compared to iterative reconstruction and FBP on image quality in CTPA performed for suspicious PE and to explore its potential for further dose reduction.

## Methods

### Patient selection and study design

This retrospective study was performed as a single-center cohort study. We analyzed 220 consecutive patients who had been referred to our department between November 2022 and April 2023 for a clinically indicated CT pulmonary angiography. Individuals were selected by searching the Radiology information system of our hospital for CTPA studies. Patients’ age, weight and height as well as radiation metrics were documented. For 8 patients, weight and height data were missing.

### Ethical approval and informed consent

The study was approved by the responsible Institutional Review Board (Ethical committee, Rostock University Medical Center) with waiver of informed consent and was conducted in accordance with the Declaration of Helsinki.

### CT acquisition protocol

All patients were examined on a 256-detector-row CT system (Revolution CT, GE HealthCare) with a gantry rotation time of 0.28 s/rotation, a tube voltage of 100 kV and an attenuation-based automatic tube current with a reference noise index of 15. A bolus of intravenous contrast agent (60–70 ml of Imeron® 400 mg/ml or Ultravist® 370 mg/ml) was injected with a flow of 4.0 ml/s followed by a saline chaser at the same injection rate. Bolus tracking technique initiated the scan after achieving a determined threshold in the main pulmonary artery. The volumetric CT dose index (CTDI_vol_) and the dose length product (DLP) were collected from the dose reports in the picture archiving and communication system. All CT acquisition parameter are summarized in Table 1.

**Table 1:** CT protocol.

Parameter	Value
**Acquisition parameters**	
Tube voltage [kV]	100
Tube current	Attenuation-based automatic tube current
Reference noise index	Reference noise index of 15
**Contrast protocol**	
Contrast volume [mL]	60 (60–70)
Contrast concentration [mg/mL]	370–400
Flow rate [mL/s]	4
Saline chaser [mL]	40 at 4 mL/s
**Radiation metrics**	
CTDI_vol_ [mGy]	3.1 (2.6–3.4)
DLP [mGy*cm]	111 (93–125)

### Image reconstruction

For every patient, seven axial image series were reconstructed using filtered back projection (FBP), hybrid iterative reconstruction (ASiR-V (GE HealthCare) with 30%, 60% and 90% intensity) and DLIR (TrueFidelity™ (GE HealthCare). DLIR was applied in low (DLIR-L), medium (DLIR-M) and high (DLIR-H) strength. Slice thickness was 0.625 mm with a slice interval of 0.25 mm for all reconstructed images. Reconstruction time was approximately 25 frames per second for ASiR-V and approximately 10 frames per second for DLIR.

### Analysis of objective image quality

**Figure 1:**
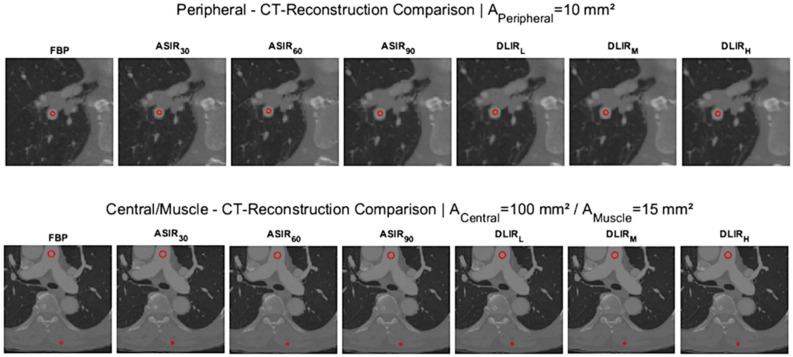
Visualization of objective image measurement: examples with one circular ROI (segmental pulmonary artery—peripheral) in upper row and two circular ROIs (main pulmonary artery—central and muscle) in lower row, which were defined at the same position for all reconstructed images (FBP; ASiR-V 30%, 60% and 90%; DLIR in low (L), medium (M) or high (H) strengths) per patient.

Quantitative analysis of image quality was performed by determining intravascular attenuation, intravascular image noise, signal-to-noise ratio (SNR) and contrast-to-noise ratio (CNR). For each patient three circular region of interests (ROI) where positioned in the reconstructed CT images. The first ROI (${\varvec{R}}_{Central} )$ was positioned in the main pulmonary artery with an area of 100 mm^2^, the second ROI $\left( {{\varvec{R}}_{periph} } \right)$ was positioned in one segmental pulmonary artery with an area of 10 mm^2^ and the third ROI $({\varvec{R}}_{Muscle} )$ was positioned in the paraspinal muscle with an area of 15 mm^2^ for determining the reference attenuation. All ROIs were systematically applied to the DICOM image data in all reconstructions (see Fig. 1). Attention was paid to avoid artifacts and contrast filling defects in the presence of PE. Manual ROI positioning was only done in one of the seven reconstructed images and the ROIs were automatically transferred into the remaining reconstructed axial series of each patient at the exact same slice position. All measurements of attenuation and image noise were extracted from those ROIs using an analyzer tool developed in house based on Matlab R2022a—Update 4. The detailed determination of the SNR and CNR including an image example can be found in the supplemental section “Determination of signal-to-noise ratio (SNR) and contrast-to-noise ratio (CNR)”.

Intravascular image noise was defined as the standard deviation of the intravascular CT attenuation. Paraspinal muscles were used for attenuation references. Signal-to-noise ratio and contrast-to-noise ratio were computed for each patient in main pulmonary artery and in segmental pulmonary artery by using the equation below*.*

$$SNR_{central} = \frac{{S_{central} }}{{N_{central} }}\;\;{\text{and}}\;\;SNR_{peripheral} = \frac{{S_{peripheral} }}{{N_{peripheral} }}$$

$$CNR_{central} = \frac{{|S_{central} - S_{muscle} |}}{{N_{central} }}\;\;{\text{and}}\;\;CNR_{peripheral} = \frac{{|S_{peripheral} - S_{muscle} |}}{{N_{peripheral} }},$$

where $S_{central}$ is the intravascular attenuation [HU], $S_{peripheral}$ is the segmental pulmonary artery attenuation [HU], $S_{muscle}$ is the attenuation in paraspinal muscle [HU] and $N_{central}$ is the intravascular image noise [HU] and $N_{peripheral}$ is the segmental pulmonary artery image noise [HU] as well.

These four features, where determined for all CT reconstructions (FBP, ASiR-V 30%, ASiR-V 60% and ASiR-V 90%, DLIR-L, DLIR-M and DLIR-H) and analyzed for all patients in the subsection “Statistical analysis”.

To evaluate the specific effect of DLIR depending on body size, objective image quality was further analyzed by dividing the patient cohort into three subgroups based on BMI: normal weight (BMI < 25 kg/m^2^), overweight (25.0–29.9 kg/m^2^) and obese (≥ 30 kg/m^2^).

For the subset of patients with confirmed PE, we additionally quantified the PE-specific contrast-to-noise ratio defined as

$$CNR_{PE} = \frac{{|S_{intra} - S_{PE} |}}{{N_{intra} }},$$

where $S_{intra}$ is the intravascular attenuation adjacent to pulmonary embolus [HU], $S_{PE}$ is the attenuation within pulmonary embolism [HU] and $N_{intra}$ is the intravascular image noise in [HU].

### Analysis of subjective image quality

Subjective image quality was assessed independently by two experienced radiologists (both 5 years of experience). Both observers rated the image quality on a 5-point scale (5 = excellent; 4 = good; 3 = sufficient; 2 = poor; 1 = non-diagnostic). Separate ratings were made for central and peripheral pulmonary arteries.

### Statistical analysis

GraphPad Prism (Version 9.5.1, GraphPad Software LLC) was used for statistical analysis. All results are presented as median and interquartile range (25th–75th percentiles). Demographic parameters and results of CTPA were compared between men and women using Mann–Whitney-U test and Chi-squared test as appropriate. Friedman test for paired data was used to compare objective image quality. Dunn's test for pairwise multiple comparisons of the ranked data was used for post-hoc test following Friedman test. P-values < 0.05 were considered as statistically significant.

## Results

### Patient characteristics, radiation dose and results of CTPA

Patient characteristics are summarized in Table 2. CTPA examinations of 220 patients (116 male, 104 female) with a median age of 70 years (IQR: 58–80 years) were included into the study. Median BMI was 26.4 kg/m^2^ (IQR: 23.5–30.2 kg/m^2^). Median CTDI was 3.1 mGy (IQR: 2.6–3.4 mGy) and DLP was 111 mGy*cm (IQR: 93–125 mGy*cm, Table 1). Overall, 40 patients (18.2%) had a CTPA positive for pulmonary embolism. There was a non-significant trend for a higher diagnostic yield of CTPA in women (21.2% positive for PE) than in men (15.5%, p = 0.28). PE was anatomically categorized by the most central location of emboli into central (n = 15), lobar (n = 8), segmental (n = 14) and subsegmental (n = 3).

**Table 2:** Patient Characteristics.

	All patients (n = 220)	Men (n = 116)	Women (n = 104)	p-value
**Demographic parameters**				
Age	70 (58–80)	69 (58–78)	71 (58–82)	0.18
Weight [kg]	78 (67–90)	84 (73–95)	71 (63–82)	< 0.05
Height [m]	1.70 (1.65–1.79)	1.78 (1.72–1.82)	1.65 (1.60–1.70)	< 0.05
BMI [kg/m^2^]	26.4 (23.5–30.2)	26.9 (23.9–29.4)	25.8 (23.4–32.0)	0.85
**Result of CTPA**				
No PE	180	98	82	0.28
PE	40	18	22	
Central	15	5	10	
Lobar	8	5	3	
Segmental	14	8	6	
Subsegmental	3	0	3	

### Objective image quality

**Figure 2:**
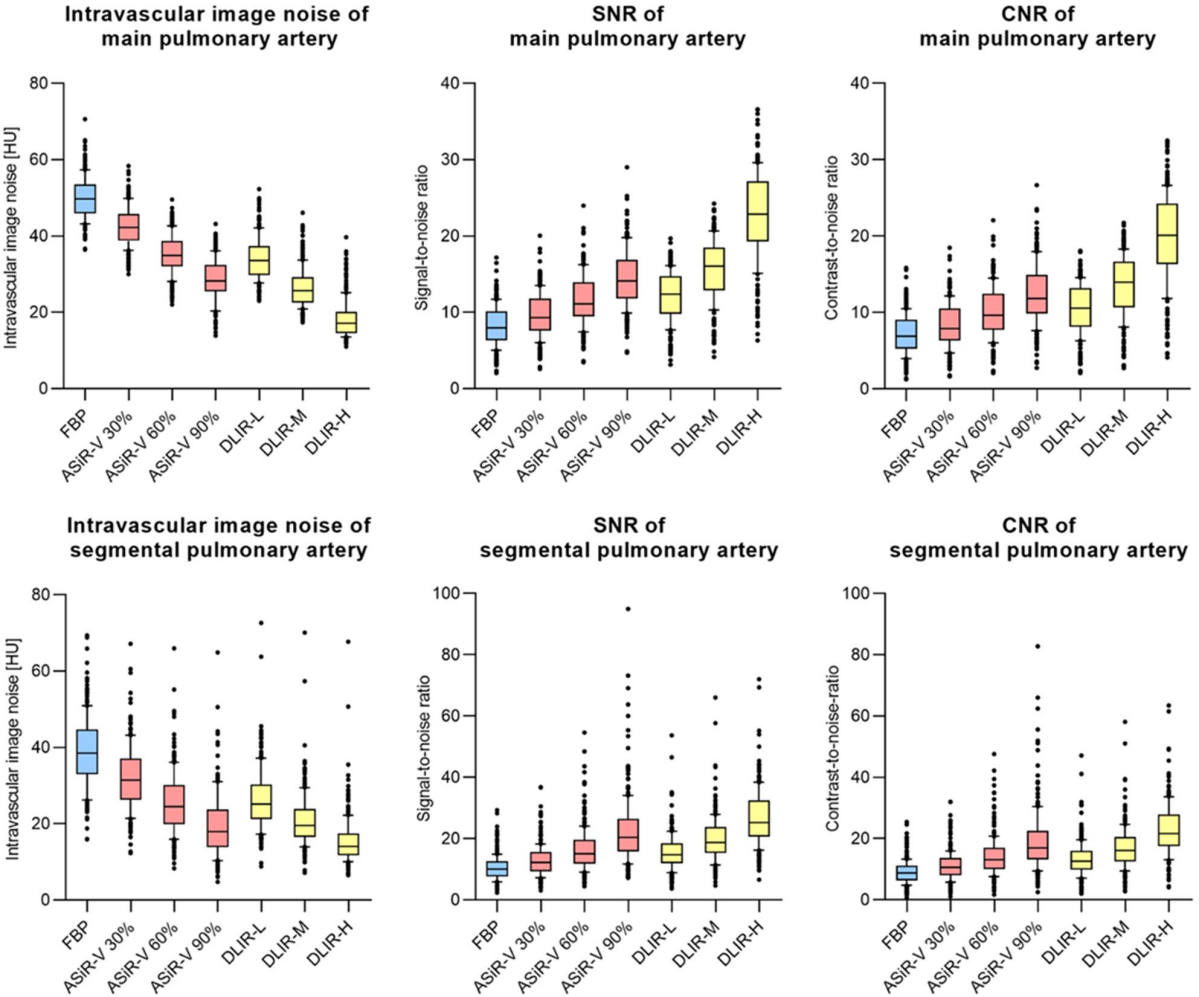
Objective image quality: intravascular image noise (in HU), SNR and CNR of main pulmonary artery and segmental pulmonary artery. Blue boxplots indicate values of filtered back projection (FBP), red boxplots values of adaptive statistical iterative reconstruction (ASiR-V) in different levels and yellow boxplots indicate results of deep learning-based image reconstruction (DLIR) in different levels.

The results of the objective image quality analysis can be found in Table 3 and are visualized in Fig. 2. Differences in intravascular attenuation of the main pulmonary artery were minimal between reconstruction techniques. Image noise was highest for FBP (median 50 HU) and decreased with increasing levels of ASiR-V and, even more so in DLIR. DLIR-H showed lowest intravascular image noise with a median value of 17 HU (IQR: 15–20 HU). For SNR, the highest values were calculated for DLIR-H, DLIR-M and ASiR-V 90% with 23 HU (IQR: 19–27 HU), 16 HU (IQR: 13–18 HU) and 14 HU (IQR: 12–17 HU), respectively. CNR showed a similar trend.

**Table 3:** Objective image quality.

	FBP	ASiR-V			DLIR			p-value
		30%	60%	90%	Low	Medium	High	
Attenuation paraspinal muscle [HU]	55 (46–62)	55 (47–61)	54 (47–61)	54 (47–60)	54 (48–60)	53 (48–59)	53 (49–58)	< 0.05
Main pulmonary artery								
Intravascular attenuation [HU]	399 (312–494)	399 (311–494)	399 (311–494)	398 (311–493)	399 (312–495)	400 (312–495)	400 (312–495)	< 0.05
Intravascular image noise [HU]	50 (46–54)	42 (39–46)	35 (32–39)	28 (25–32)	34 (30–37)	26 (23–29)	17 (15–20)	< 0.05
Signal-to-Noise ratio	8 (6–10)	9 (8–12)	11 (9–14)	14 (12–17)	12 (10–15)	16 (13–18)	23 (19–27)	< 0.05
Contrast-to-Noise ratio	7 (5–9)	8 (6–11)	10 (8–12)	12 (10–15)	11 (8–13)	14 (11–17)	20 (16–24)	< 0.05
Segmental pulmonary artery								
Intravascular attenuation [HU]	374 (307–461)	374 (305–461)	373 (303–461)	372 (301–461)	371 (306–461)	371 (304–461)	370 (301–461)	< 0.05
Intravascular image noise [HU]	38 (33–45)	31 (26–37)	25 (20–30)	18 (14–24)	25 (21–30)	20 (17–24)	14 (12–17)	< 0.05
Signal-to-Noise ratio	10 (8–13)	12 (9–16)	15 (12–20)	20 (16–27)	15 (12–18)	19 (15–24)	25 (21–33)	< 0.05
Contrast-to-Noise ratio	9 (6–11)	11 (8–14)	13 (10–17)	17 (13–23)	13 (10–16)	16 (13–21)	22 (17–28)	< 0.05

Intravascular image noise was lower and SNR and CNR were higher in segmental pulmonary arteries compared to the main pulmonary artery. The effects of ASiR-V and DLIR on image noise, SNR and CNR were consistent between main pulmonary artery and segmental pulmonary arteries. DLIR-L showed similar image quality as ASiR-V with an intensity of 60% and DLIR-M was equivalent to ASiR-V 90%. These were the only pairwise comparisons, which showed no statistically significant differences.

DLIR-H provided a substantial additional improvement in contrast-to-noise ratio by 29–67% compared to ASiR-V 90% (20 (IQR: 16–24) vs. 12 (IQR: 10–15) for main and 22 (IQR: 17–28) vs. 17 (IQR: 13–23) for segmental pulmonary arteries, p < 0.05). P-values of all pairwise comparisons of CNR can be found in Supplementary Tables S1–S2.

#### Objective image quality stratified by body size

**Figure 3:**
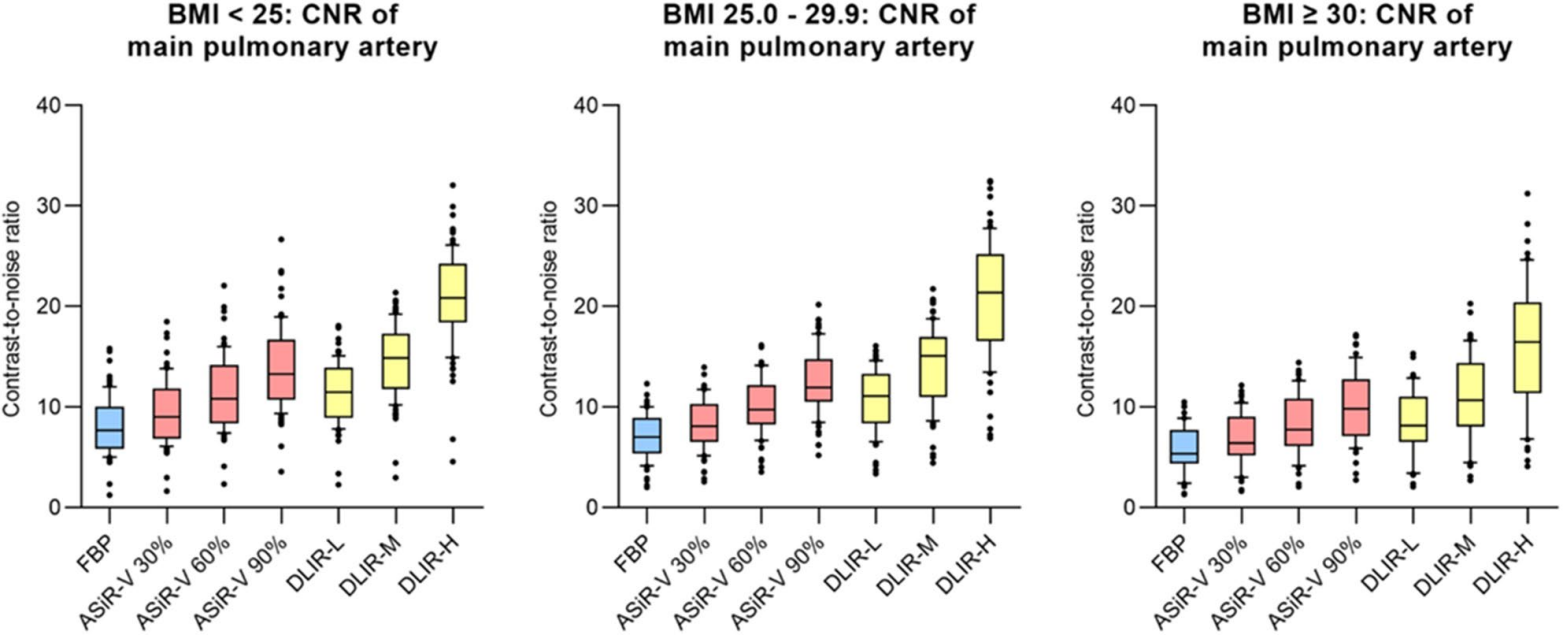
Objective image quality adjusted by BMI: CNR of main pulmonary artery in different BMI groups. Blue boxplots indicate values of filtered back projection (FBP), red boxplots values of adaptive statistical iterative reconstruction (ASiR-V) in different levels and yellow boxplots indicate results of deep learning-based image reconstruction (DLIR) in different levels.

The results of the subgroup analysis based on patients’ body size are shown in Fig. 3 (for main pulmonary artery) and Table 4. Supplementary Fig. S1 shows the corresponding subgroup analysis for the segmental pulmonary artery. Intravascular attenuation was lower in heavier patients while image noise did not show major differences due to the use of attenuation-based tube current modulation. The observed effects of ASiR-V and DLIR on image quality were consistent across all subgroups. For all subgroups, SNR and CNR were highest for DLIR-H and lowest for FBP. In all subgroups, image quality parameters were equivalent between DLIR-L and ASiR-V 60% as well as between DLIR-M and ASiR-V 90%.

**Table 4:** Objective image quality: subgroup analysis by body size (main pulmonary artery).

	FBP	ASiR-V			DLIR			p-value
		30%	60%	90%	Low	Medium	High	
Intravascular attenuation [HU]								
BMI < 25 (n = 85)	451 (356–568)	451 (356–569)	450 (355–569)	450 (355–569)	451 (357–568)	451 (356–568)	451 (356–568)	< 0.05
BMI 25.0–29.9 (n = 73)	388 (323–481)	388 (324–480)	388 (324–480)	389 (324–480)	389 (325–480)	390 (326–480)	389 (326–481)	< 0.05
BMI ≥ 30 (n = 54)	333 (267–440)	334 (267–440)	334 (267–440)	334 (267–439)	332 (267–439)	332 (267–439)	332 (267–438)	< 0.05
Intravascular image noise [HU]								
BMI < 25 (n = 85)	50 (47–55)	43 (39–47)	35 (33–39)	29 (26–33)	35 (30–39)	27 (23–30)	18 (15–21)	< 0.05
BMI 25.0–29.9 (n = 73)	48 (45–51)	41 (38–44)	33 (31–37)	27 (25–31)	31 (28–35)	24 (21–27)	16 (14–18)	< 0.05
BMI ≥ 30 (n = 54)	52 (46–55)	44 (39–47)	35 (32–40)	28 (24–34)	36 (30–39)	27 (23–30)	17 (15–20)	< 0.05
Signal-to-noise ratio								
BMI < 25 (n = 85)	9 (7–11)	10 (8–13)	12 (10–15)	15 (13–18)	13 (11–15)	17 (14–19)	24 (21–27)	< 0.05
BMI 25.0–29.9 (n = 73)	8 (7–10)	10 (8–12)	11 (10–14)	14 (12–17)	13 (10–15)	17 (14–19)	25 (20–29)	< 0.05
BMI ≥ 30 (n = 54)	6 (5–9)	8 (6–10)	9 (7–12)	12 (10–15)	10 (8–13)	13 (10–16)	19 (14–23)	< 0.05
Contrast-to-noise ratio								
BMI < 25 (n = 85)	8 (6–10)	9 (7–12)	11 (8–14)	13 (11–17)	12 (9–14)	15 (12–17)	21 (18–24)	< 0.05
BMI 25.0–29.9 (n = 73)	7 (5–9)	8 (7–10)	10 (8–12)	12 (11–15)	11 (8–13)	15 (11–17)	21 (17–25)	< 0.05
BMI ≥ 30 (n = 54)	5 (4–8)	6 (5–9)	8 (6–11)	10 (7–13)	8 (7–11)	11 (8–14)	17 (11–20)	< 0.05

DLIR-H provided a substantial additional improvement in contrast-to-noise ratio compared to ASiR-V 90% in all subgroups (by 62–75% for the main pulmonary artery and by 27–33% for the segmental pulmonary arteries). The results of objective image quality stratified by body size can be found in Table 4 for the main pulmonary artery and in Supplementary Table S3 for segmental pulmonary arteries. P-values for pairwise comparisons of CNR in central pulmonary arteries for all subgroups are listed in Supplementary Tables S4–S6.

#### PE-specific contrast-to-noise ratio

**Figure 4:**
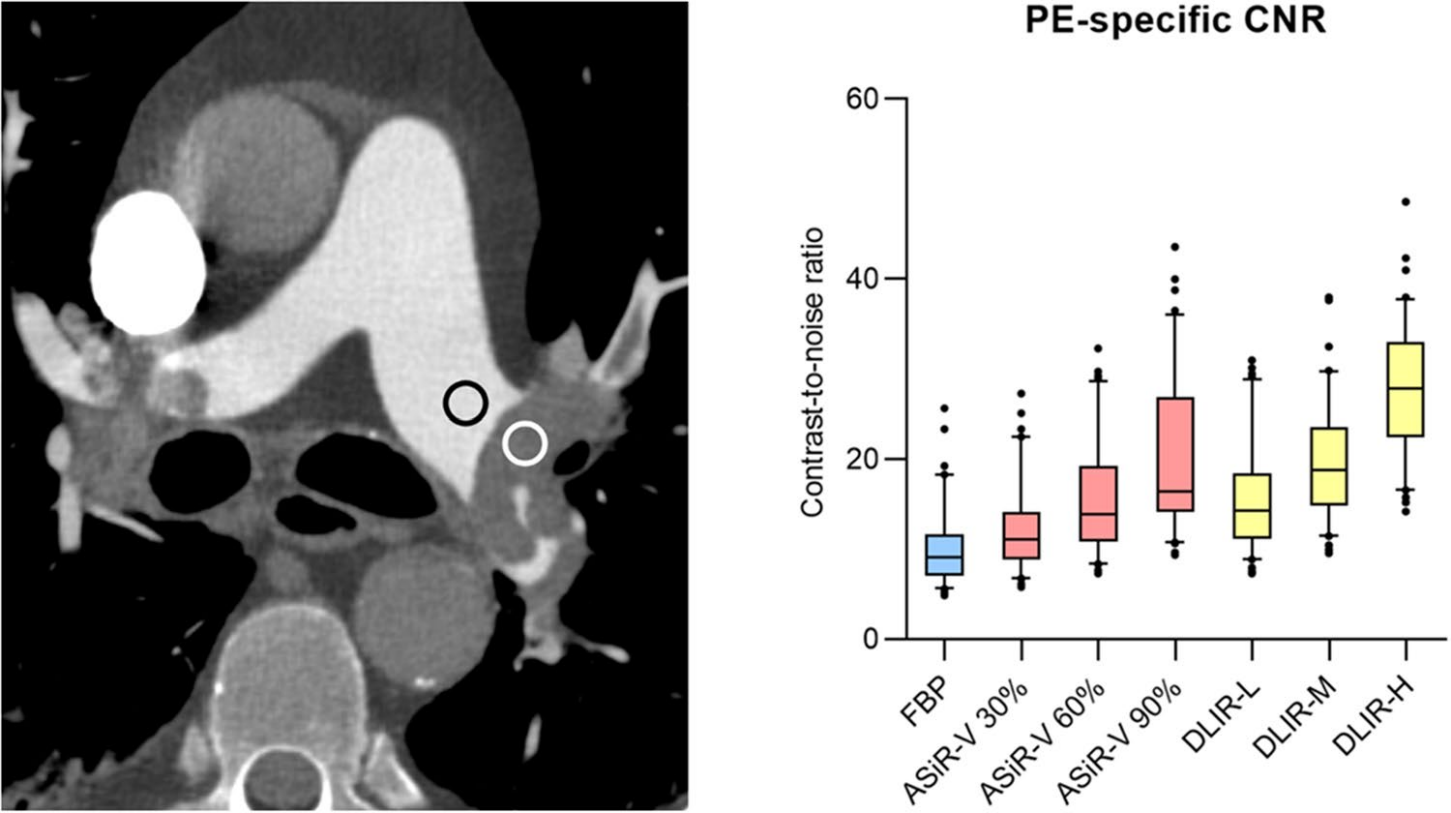
Assessment of PE-specific contrast-to-noise-ratio: for the subgroup of patients with PE (n = 40), PE-specific contrast-to-noise ratio quantified as intravascular attenuation adjacent to pulmonary embolus—attenuation within pulmonary embolus/intravascular image noise.

In the subgroup of 40 patients with confirmed PE, the PE-specific CNR (based on the contrast between pulmonary emboli and adjacent blood) was highest for DLIR-H with an additional increase of 75% compared to ASiR-V 90% (Fig. 4, Supplementary Table S7).

### Subjective image quality

**Figure 5:**
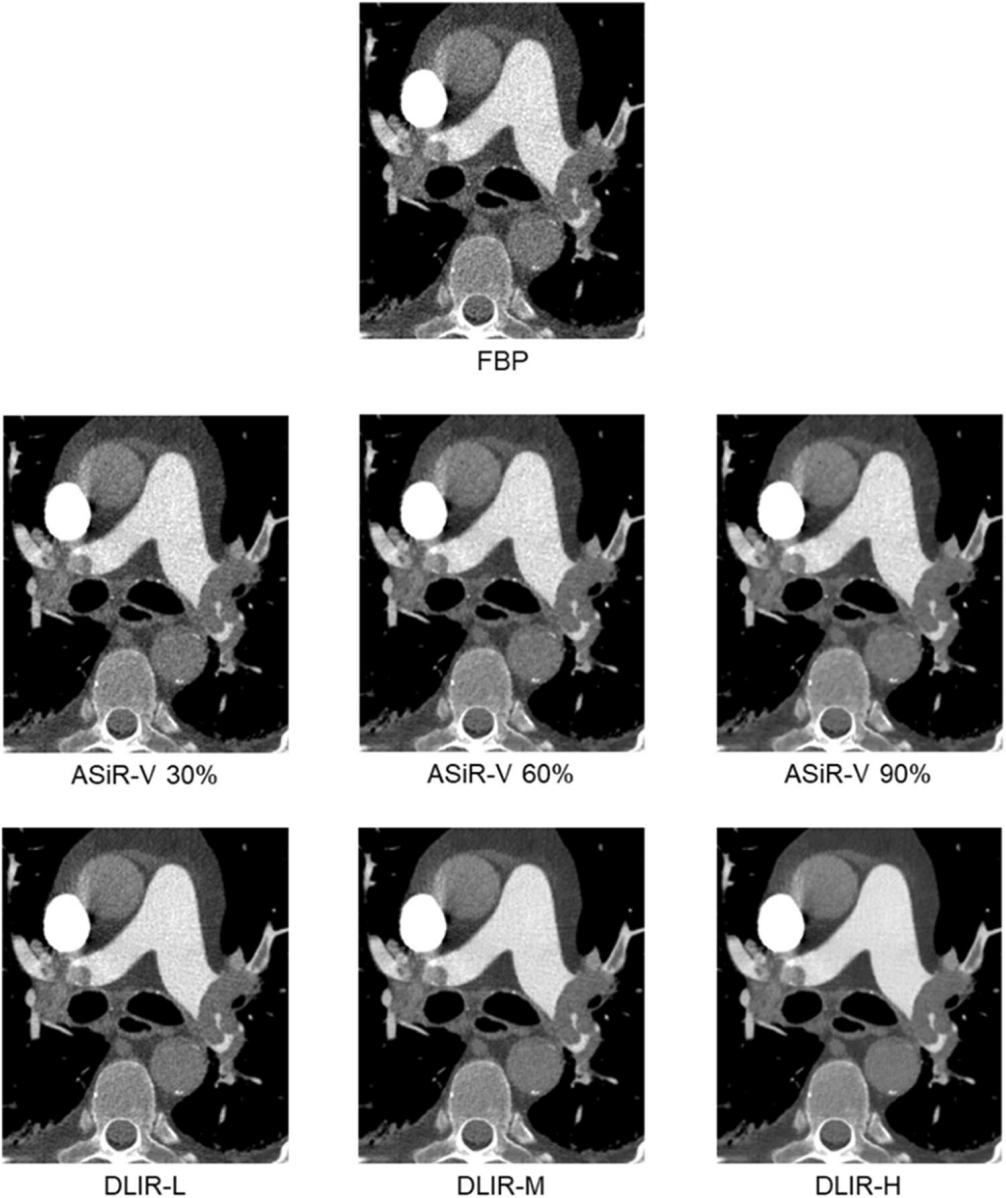
Representative image examples: CTPA images of a 70 year old man with central pulmonary embolism with different reconstruction: Filtered back projection (FBP), adaptive statistical iterative reconstruction (ASiR-V 30%, 60% and 90%) and deep learning-based image reconstruction (DLIR) in low (L), medium (M) or high (H) strengths. Window levels are identical for all reconstructions. Images were cropped to show central pulmonary vasculature.

Overall image quality ranged from sufficient to excellent. None of the studies was rated as non-diagnostic or poor quality. Subjective image quality was rated best for DLIR-M and DLIR-H, both received significantly higher image quality ratings than ASiR-V 90% (p < 0.05 for both). Subjective image quality for DLIR-L was comparable to ASiR-V 90% (p > 0.99). Detailed results of the subjective image quality assessment are displayed in Table 5. P-values for pairwise comparisons of subjective image quality are listed in Supplementary Tables S8–S9. Representative examples of the image reconstructions in a patient with central pulmonary embolism are shown in Fig. 5.

**Table 5:** Subjective image quality.

	FBP	ASiR-V			DLIR			p-value
		30%	60%	90%	Low	Medium	High	
Main pulmonary artery								
Reader 1	4 (3–4)	4 (3–4)	4 (4–5)	5 (4–5)	5 (5–5)	5 (5–5)	5 (5–5)	< 0.05
Reader 2	3 (3–3)	3 (3–3)	4 (3–4)	4 (3–4)	4 (4–4)	5 (4–5)	5 (5–5)	< 0.05
Median (Range)	3 (3–4)	3 (3–4)	4 (3–4)	4 (4–5)	4 (4–5)	5 (4–5)	5 (5–5)	< 0.05
Segmental pulmonary artery								
Reader 1	4 (3–4)	4 (3–4)	4 (4–5)	5 (4–5)	5 (4–5)	5 (5–5)	5 (5–5)	< 0.05
Reader 2	3 (3–3)	3 (3–3)	4 (3–4)	4 (3–4)	4 (4–4)	5 (4–5)	5 (5–5)	< 0.05
Median (Range)	3 (3–4)	3 (3–4)	4 (3–4)	4 (3–5)	4 (4–5)	5 (4–5)	5 (5–5)	< 0.05

## Discussion

In our study, we compared different CT image reconstructions (FBP, ASiR-V and DLIR) of CTPA in patients suspicious for PE. By analyzing the objective and subjective image quality, we found major improvements in diagnostic confidence, SNR and CNR in deep learning reconstructed images in high strength. Even though ASiR-V reconstructions with 60% and 90% intensity were not significantly better than DLIR in low and medium strength, respectively, DLIR in high strength showed improved image quality even in adipose patients.

To our knowledge, only one study previously investigated DLIR for CTPA^29^. This previous study investigated the DLIR algorithm of a different vendor at 50% strength and compared this to the same vendor’s hybrid iterative reconstruction technique at 50% strength. It could be demonstrated that subjective and objective image quality was improved (17% improvement in CNR) with DLIR even though radiation dose was reduced by 17% in the DLIR group. The results of our study cannot be directly compared to this previous study since our study was performed using the DLIR algorithm of a different vendor and a different study design. We also systematically investigated the effect of DLIR compared to iterative reconstruction using different strengths of both reconstruction techniques in identical patients (intra-individual comparison).

Furthermore, in our study, we found that DLIR-H provided a substantial additional improvement in contrast-to-noise ratio by 29–67% compared to ASiR-V 90% driven by an additional reduction in image noise by 22–39%. Image noise is inversely proportional to the square root of the applied radiation dose in linear image processing. Even though DLIR is a non-linear image processing method, a further dose reduction compared to ASiR-V 90% can be anticipated. Despite possible dose reductions, the dose levels should always be established based on the specific clinical task to ensure diagnostic accuracy. Therefore, further studies are needed to confirm that image quality and diagnostic confidence are indeed preserved at such low radiation dose.

We also observed statistically significant differences in intravascular attenuation between reconstruction techniques but these were minimal (≤ 4 HU, approximately 1%) and not clinically relevant. This is due to the high sensitivity of the paired statistical tests in which even minimal differences can be statistically significant if they are consistent across individuals.

One interesting aspect of our data is that the relative benefit of DLIR compared to ASiR-V was even greater for subjective image quality than for objective image quality. Specifically, while objective quality was equivalent between DLIR-M and ASiR-V 90%, subjective image quality was rated as significantly better in DLIR-M compared to ASiR-V 90%. The most likely explanation for this finding is that, at equivalent levels of noise reduction, DLIR images are perceived by radiologists as more “natural” and less “plastic” compared to images generated with iterative reconstruction. This has also been observed in other studies on DLIR^17^.

We applied DLIR to CTPA studies in suspected PE. Our results are in line with those that have been described for various cardiovascular CT applications including coronary CTA^19–23^, CT for planning of transcatheter aortic valve repair^24,25^, CT of the aorta^26^, head and neck CT angiography^27^ and triple-rule-out CT^28^. In the field of thoracic imaging, several previous studies have investigated the value of DLIR with regards to pulmonary imaging and lung nodule detection. Specifically, one prospective study found that DLIR applied to ultra-low dose CT improved the nodule detection rate and the accuracy of nodule measurements compared to ASiR-V reconstructions^30^. A phantom study demonstrated higher accuracy of ultra-low-dose chest with DLIR for volumetric assessment of ground glass nodules compared to model-based iterative reconstruction and hybrid iterative reconstruction^31^. A study on cadaveric human lungs reported that with DLIR dose could be reduced by up to 85% with preserved image quality compared to FBP^32^. Others have used combinations of deep learning-based image denoising and iterative reconstruction and reported improved assessment of pulmonary nodules when both were combined^33^.

Our study has several limitations. Due to the retrospective nature of our investigation, we were not able to directly demonstrate that DLIR allows for further reduction in radiation dose with constant image quality. We evaluated a specific DLIR algorithm of a single manufacturer. Further, we did not evaluate the impact of DLIR on diagnostic accuracy of CTPA or its impact on the performance of automated PE detection algorithms.

In conclusions, our analysis demonstrated that DLIR significantly outperforms iterative reconstruction for increasing objective and subjective image quality in CT pulmonary angiography. This may allow for further reductions in radiation exposure in suspected pulmonary embolism.

### Supplementary Information

Supplementary Information 1.

Supplementary Information 2.

## Data Availability

The dataset generated and analyzed during the current study are available from the corresponding author on reasonable request.

## Abbreviations

ASiR-V: Adaptive statistical iterative reconstruction
CNR: Contrast-to-noise ratio
CT: Computed tomography
CTA: Computed tomography angiography
CTDI_vol_: Volume computed tomography dose index
CTPA: Computed tomography pulmonary angiography
DLIR: Deep learning-based image reconstruction
DLP: Dose length product
FBP: Filtered back projection
HU: Hounsfield units
PE: Pulmonary embolism
ROI: Region of interest
SNR: Signal-to-noise ratio
