# Supplementary material for: Improved image quality in CT pulmonary angiography using deep learning-based image reconstruction
Source: Sci Rep. 2024 Jan 30;14:2494. doi: 10.1038/s41598-024-52517-2 (PMC10827738; doi:10.1038/s41598-024-52517-2)
Supplement: Supplementary file 1 — Supplementary Information 1. [file 41598_2024_52517_MOESM1_ESM.pdf]

Improved Image Quality in CT Pulmonary Angiography using Deep Learning-based Image Reconstruction

Ann-Christin Klemenz, PhD[1], Lasse Albrecht[1], Mathias Manzke, MSc[1],

Antonia Dalmer, MD[1], Benjamin Böttcher, MD[1], Alexey Surov, MD[2]

Marc-André Weber, MD MSc[1], Felix G. Meinel, MD[1]

[1] Institute of Diagnostic and Interventional Radiology, Pediatric Radiology and Neuroradiology, University Medical Centre Rostock, Germany

[2] Department of Radiology, Mühlenkreiskliniken Minden, Ruhr-University Bochum, Germany.

Original Research

Short title: DLIR for CT Pulmonary Angiography

Keywords

pulmonary embolism, deep learning, image reconstruction, image quality, computed tomography

Address for correspondence:

Felix G. Meinel, M.D.

Institute for Diagnostic and Interventional Radiology,

Pediatric Radiology and Neuroradiology

University Medical Centre Rostock

Schillingallee 36

18057 Rostock, Germany

Phone +49 381 494 9275

Fax +49 381 494 9202

The authors have no conflicts of interest to declare.

Supplemental Section

"Determination of signal-to-noise ratio (SNR) and contrast-to-noise ratio (CNR)"

For a given center point $(x_m, y_m)^T \in \boldsymbol{P} \subset \mathbb{N}^2$ and a given area A, a ROI $\boldsymbol{R}$ is defined as the set of all points $(x, y)^T \in \boldsymbol{P}$ in a DICOM image which can be located within the circle boundary radius r:

$$\boldsymbol{R} = \{(x, y)^T \in \boldsymbol{P} : (x - x_m)^2 + (y - y_m)^2 \leq r^2\}.$$

Particularly, for the calculation of the ROIs, the pixels positions were transformed into polar coordinates so that we can apply the relationship:

$$\begin{pmatrix} x \\ y \end{pmatrix} = \begin{pmatrix} r \cdot cos\varphi + x_m \\ r \cdot sin\varphi + y_m \end{pmatrix}, \varphi \in [0,2\pi].$$

The constant value $A = const.$ $[mm^2]$ was defined for all measurements to ensure that each ROI is completely located within the pulmonary artery and the paraspinal muscle.

From the ROI image points obtained in this way, the attenuation distributions of the ROI pixels were always examined in pairs and the signal intensity S and the noise N were calculated under usage of the arithmetic mean and the standard deviation.

Intravascular image noise was defined as the standard deviation of the intravascular CT attenuation. Paraspinal muscles were used for attenuation references.

Signal-to-noise ratios were computed for each patient in main pulmonary artery and in segmental pulmonary artery by using the equations below:

$$SNR_{central} = \frac{S_{central}}{N_{central}} \text{ and } SNR_{peripheral} = \frac{S_{peripheral}}{N_{peripheral}}$$

and the contrast-to-noise ratios with

$$CNR_{central} = \frac{|S_{central} - S_{muscle}|}{N_{central}} \text{ and } CNR_{peripheral} = \frac{|S_{peripheral} - S_{muscle}|}{N_{peripheral}},$$

where $S_{central}$ is the intravascular attenuation [HU], $S_{peripheral}$ is the segmental pulmonary artery attenuation [HU], S_{muscle} is the attenuation in paraspinal muscle [HU] and $N_{central}$ is the intravascular image noise [HU] and $N_{peripheral}$ is the segmental pulmonary artery image noise [HU] as well.

These four features, where determined for all CT reconstructions (FBP, ASiR-V 30%, ASiR-V 60% and ASiR-V 90%, DLIR-L, DLIR-M and DLIR-H) and analyzed for all patients in the subsection statistical analysis.
