# Supplementary material for: Improved image quality in CT pulmonary angiography using deep learning-based image reconstruction
Source: Sci Rep. 2024 Jan 30;14:2494. doi: 10.1038/s41598-024-52517-2 (PMC10827738; doi:10.1038/s41598-024-52517-2)

Improved Image Quality in CT Pulmonary Angiography using Deep Learning-based Image Reconstruction

Ann-Christin Klemenz, PhD[1], Lasse Albrecht[1], Mathias Manzke, MSc[1],

Antonia Dalmer, MD[1], Benjamin Böttcher, MD[1], Alexey Surov, MD[2]

Marc-André Weber, MD MSc[1], Felix G. Meinel, MD[1]

[1] Institute of Diagnostic and Interventional Radiology, Pediatric Radiology and Neuroradiology, University Medical Centre Rostock, Germany

[2] Department of Radiology, Mühlenkreiskliniken Minden, Ruhr-University Bochum, Germany.

Original Research

Short title: DLIR for CT Pulmonary Angiography

Keywords

pulmonary embolism, deep learning, image reconstruction, image quality, computed tomography

Address for correspondence:

Felix G. Meinel, M.D.

Institute for Diagnostic and Interventional Radiology,

Pediatric Radiology and Neuroradiology

University Medical Centre Rostock

Schillingallee 36

18057 Rostock, Germany

Phone +49 381 494 9275

Fax +49 381 494 9202

The authors have no conflicts of interest to declare.

SUPPLEMENTARY MATERIAL

Supplementary Table S1: P-Values for pairwise comparisons of CNR (main pulmonary artery).

	FBP	ASiR-V 30%	ASiR-V 60%	ASiR-V 90%	DLIR-L	DLIR-M	DLIR-H
**FBP**	-	-	-	-	-	-	-
**ASiR-V 30%**	<0.05	-	-	-	-	-	-
**ASiR-V 60%**	<0.05	<0.05	-	-	-	-	-
**ASiR-V 90%**	<0.05	<0.05	<0.05	-	-	-	-
**DLIR-L**	<0.05	<0.05	0.99	<0.05	-	-	-
**DLIR-M**	<0.05	<0.05	<0.05	0.51	<0.05	-	-
**DLIR-H**	<0.05	<0.05	<0.05	<0.05	<0.05	<0.05	-

Supplementary Table S2: P-Values for pairwise comparisons of CNR (segmental pulmonary artery).

	FBP	ASiR-V 30%	ASiR-V 60%	ASiR-V 90%	DLIR-L	DLIR-M	DLIR-H
**FBP**	-	-	-	-	-	-	-
**ASiR-V 30%**	<0.05	-	-	-	-	-	-
**ASiR-V 60%**	<0.05	<0.05	-	-	-	-	-
**ASiR-V 90%**	<0.05	<0.05	<0.05	-	-	-	-
**DLIR-L**	<0.05	<0.05	>0.99	<0.05	-	-	-
**DLIR-M**	<0.05	<0.05	<0.05	0.19	<0.05	-	-
**DLIR-H**	<0.05	<0.05	<0.05	<0.05	<0.05	<0.05	-

Supplementary Table S3: Objective Image Quality: Subgroup Analysis by Body Size (Segmental Pulmonary Artery).

	FBP	ASiR-V			DLIR			P-value
		30%	60%	90%	Low	Medium	High	
**Intravascular attenuation [HU]**								
BMI < 25 (n=85)	388 (331-520)	387 (331-522)	387 (330-523)	388 (330-525)	387 (335-523)	386 (336-524)	384 (337-524)	<0.05
BMI 25.0 - 29.9 (n=73)	373 (330-463)	373 (328-462)	372 (326-461)	372 (324-460)	372 (325-455)	371 (324-454)	371 (322-452)	<0.05
BMI ≥ 30 (n=54)	333 (263-397)	333 (263-397)	332 (262-397)	332 (261-395)	332 (263-396)	334 (263-394)	334 (263-393)	0.30
**Intravascular image noise [HU]**								
BMI < 25 (n=85)	39 (33-44)	32 (26-37)	25 (20-30)	18 (14-24)	26 (21-31)	21 (16-25)	15 (12-18)	<0.05
BMI 25.0 - 29.9 (n=73)	37 (29-44)	30 (24-36)	23 (18-29)	16 (13-23)	24 (20-27)	18 (16-22)	13 (11-16)	<0.05
BMI ≥ 30 (n=54)	40 (35-47)	33 (28-40)	25 (21-31)	19 (15-24)	26 (23-31)	20 (17-25)	15 (12-18)	<0.05
**Signal-to-noise-ratio**								
BMI < 25 (n=85)	11 (8-13)	13 (10-16)	16 (12-21)	21 (16-28)	15 (12-19)	19 (16-24)	26 (21-33)	<0.05
BMI 25.0 - 29.9 (n=73)	11 (9-13)	13 (11-16)	16 (14-21)	21 (17-30)	16 (13-19)	19 (16-25)	28 (22-35)	<0.05
BMI ≥ 30 (n=54)	9 (6-11)	10 (8-13)	13 (10-17)	18 (13-22)	12 (10-16)	16 (13-20)	23 (18-28)	<0.05
**Contrast-to-noise-ratio**								
BMI < 25 (n=85)	9 (7-12)	11 (8-14)	14 (11-19)	18 (13-24)	13 (10-17)	17 (13-21)	23 (18-29)	<0.05
BMI 25.0 - 29.9 (n=73)	9 (7-12)	11 (9-14)	14 (11-18)	18 (15-26)	14 (11-17)	17 (14-22)	24 (18-30)	<0.05
BMI ≥ 30 (n=54)	7 (5-9)	9 (6-11)	11 (8-14)	15 (10-19)	11 (8-13)	14 (10-17)	19 (14-24)	<0.05

Supplementary Table S4: P-Values for pairwise comparisons of CNR (main pulmonary artery, BMI < 25 kg/m²).

	FBP	ASiR-V 30%	ASiR-V 60%	ASiR-V 90%	DLIR-L	DLIR-M	DLIR-H
**FBP**	-	-	-	-	-	-	-
**ASiR-V 30%**	<0.05	-	-	-	-	-	-
**ASiR-V 60%**	<0.05	<0.05	-	-	-	-	-
**ASiR-V 90%**	<0.05	<0.05	<0.05	-	-	-	-
**DLIR-L**	<0.05	<0.05	>0.99	<0.05	-	-	-
**DLIR-M**	<0.05	<0.05	<0.05	>0.99	<0.05	-	-
**DLIR-H**	<0.05	<0.05	<0.05	<0.05	<0.05	<0.05	-

Supplementary Table S5: P-Values for pairwise comparisons of CNR (main pulmonary artery, BMI 25.0 - 29.9 kg/m²).

	FBP	ASiR-V 30%	ASiR-V 60%	ASiR-V 90%	DLIR-L	DLIR-M	DLIR-H
**FBP**	-	-	-	-	-	-	-
**ASiR-V 30%**	0.11	-	-	-	-	-	-
**ASiR-V 60%**	<0.05	<0.05	-	-	-	-	-
**ASiR-V 90%**	<0.05	<0.05	<0.05	-	-	-	-
**DLIR-L**	<0.05	<0.05	>0.99	<0.05	-	-	-
**DLIR-M**	<0.05	<0.05	<0.05	>0.99	<0.05	-	-
**DLIR-H**	<0.05	<0.05	<0.05	<0.05	<0.05	<0.05	-

Supplementary Table S6: P-Values for pairwise comparisons of CNR (main pulmonary artery, BMI ≥ 30 kg/m²).

	FBP	ASiR-V 30%	ASiR-V 60%	ASiR-V 90%	DLIR-L	DLIR-M	DLIR-H
**FBP**	-	-	-	-	-	-	-
**ASiR-V 30%**	0.34	-	-	-	-	-	-
**ASiR-V 60%**	<0.05	<0.05	-	-	-	-	-
**ASiR-V 90%**	<0.05	<0.05	<0.05	-	-	-	-
**DLIR-L**	<0.05	<0.05	>0.99	<0.05	-	-	-
**DLIR-M**	<0.05	<0.05	<0.05	>0.99	<0.05	-	-
**DLIR-H**	<0.05	<0.05	<0.05	<0.05	<0.05	0.05	-

Supplementary Table S7: PE-specific Contrast-to-Noise-Ratio.

	FBP	ASiR-V			DLIR			P-value
		30%	60%	90%	Low	Medium	High	
Intravascular attenuation adjacent to pulmonary embolus [HU]	383 (343-453)	383 (342-453)	385 (341-452)	387 (340-452)	382 (344-453)	382 (344-453)	381 (342-453)	<0.05
Intravascular image noise adjacent to pulmonary embolus [HU]	35 (29-43)	29 (23-37)	22 (19-30)	17 (13-24)	23 (19-29)	17 (15-22)	13 (11-15)	<0.05
Intravascular attenuation within pulmonary embolism [HU]	63 (48-76)	63 (49-74)	63 (49-73)	64 (49-74)	61 (47-75)	61 (47-74)	60 (48-74)	<0.05
PE-specific Contrast-to-Noise Ratio	9 (7-12)	11 (9-14)	14 (11-19)	16 (14-27)	14 (11-18)	19 (15-24)	28 (22-33)	<0.05

Supplementary Table S8: P-Values for pairwise comparisons of subjective image quality (central pulmonary arteries, median of both readers).

	FBP	ASiR-V 30%	ASiR-V 60%	ASiR-V 90%	DLIR-L	DLIR-M	DLIR-H
**FBP**	-	-	-	-	-	-	-
**ASiR-V 30%**	>0.99	-	-	-	-	-	-
**ASiR-V 60%**	<0.05	<0.05	-	-	-	-	-
**ASiR-V 90%**	<0.05	<0.05	>0.99	-	-	-	-
**DLIR-L**	<0.05	<0.05	0.08	>0.99	-	-	-
**DLIR-M**	<0.05	<0.05	<0.05	<0.05	<0.05	-	-
**DLIR-H**	<0.05	<0.05	<0.05	<0.05	<0.05	>0.99	-

Supplementary Table S9: P-Values for pairwise comparisons of subjective image quality (peripheral pulmonary arteries, median of both readers).

	FBP	ASiR-V 30%	ASiR-V 60%	ASiR-V 90%	DLIR-L	DLIR-M	DLIR-H
**FBP**	-	-	-	-	-	-	-
**ASiR-V 30%**	>0.99	-	-	-	-	-	-
**ASiR-V 60%**	<0.05	<0.05	-	-	-	-	-
**ASiR-V 90%**	<0.05	<0.05	>0.99	-	-	-	-
**DLIR-L**	<0.05	<0.05	<0.05	0.37	-	-	-
**DLIR-M**	<0.05	<0.05	<0.05	<0.05	0.07	-	-
**DLIR-H**	<0.05	<0.05	<0.05	<0.05	<0.05	>0.99	-

Supplementary Figure S1. Objective Image Quality by Body Size (Segmental Pulmonary Artery): CNR of segmental pulmonary artery in different BMI groups. Blue boxplots indicate values of filtered back projection (FBP), red boxplots values of adaptive statistical iterative reconstruction (ASiR-V) in different levels and yellow boxplots indicate results of deep learning-based image reconstruction (DLIR) in different levels.

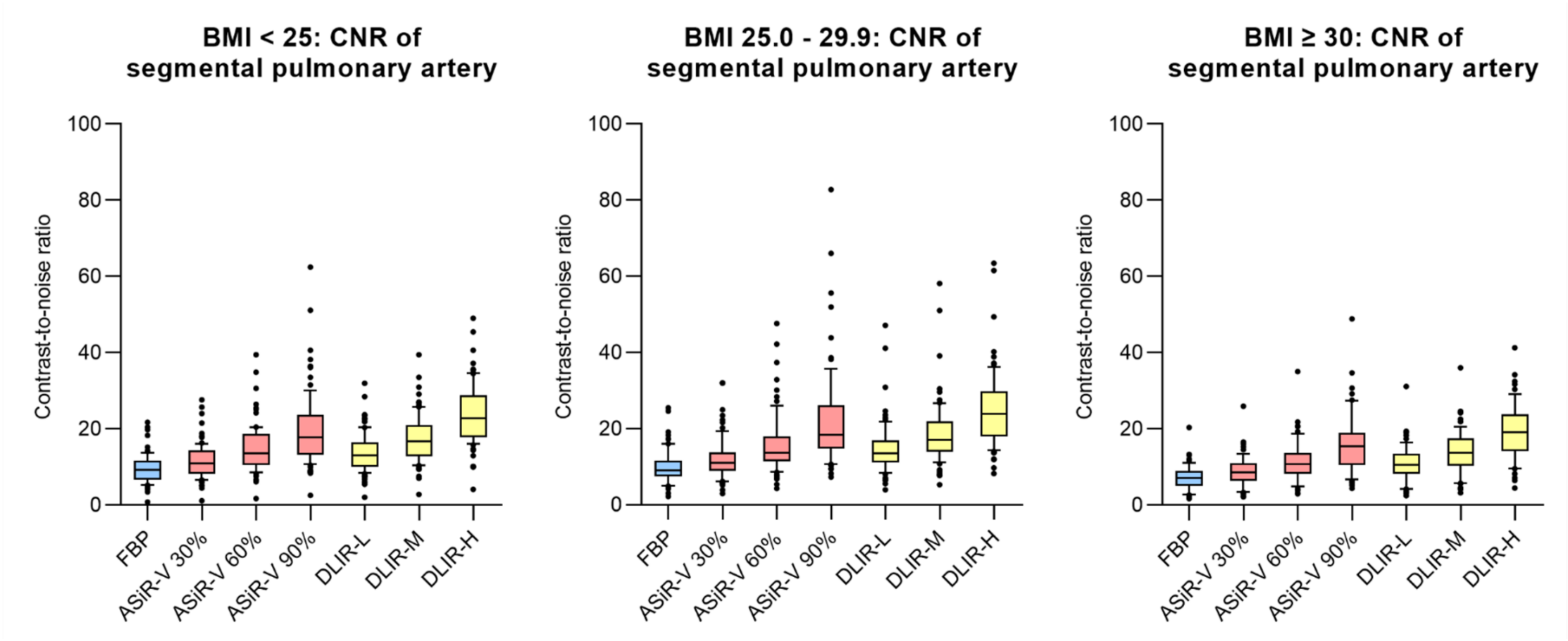

Supplement: Supplementary file 2 — Supplementary Information 2. [file 41598_2024_52517_MOESM2_ESM.pdf]
